# Relations of Tuberculosis and Scrofulosis

**Published:** 1882-07

**Authors:** 


### RELATIONS OF TUBERCULOSIS AND SCROFULOSIS.

From an experimental and clinical study of this subject, Dr. H. Martin concludes:

1. It has been proved by numerous experiments that tubercle, when inoculated, can only produce tubercle.

2. Every foreign body, whether animal or vegetable, or whether living and healthy tissue, or living pathological tissues such as that of tumors, or tissue destroyed by reagents or in process of caseous degeneration, when inoculated can only give rise to a *pseudo-tuberculosis.*

3. Scrofula, as at present understood, is perhaps not a distinct morbid process; many of its symptoms belong to the manifestations of tuberculosis. Inoculatious in series furnish the only means of reaching a positive diagnosis.

4. When this method has been applied successively to the study of the different lesions of scrofula, it will be found that they are closely associated with tuberculosis, perhaps modified by a lymphatic temperament.—*Revue de Médicine*, April 10, 1882.

